# Preoperative dexamethasone administration in hepatectomy of 25-min intermittent Pringle’s maneuver for hepatocellular carcinoma: a randomized controlled trial

**DOI:** 10.1097/JS9.0000000000000622

**Published:** 2023-07-31

**Authors:** Yang Huang, Liangliang Xu, Ning Wang, Yonggang Wei, Wentao Wang, Mingqing Xu, Li Jiang

**Affiliations:** Department of General Surgery, Division of Liver Surgery, West China Hospital, Sichuan University, Chengdu, Sichuan Province, People’s Republic of China

**Keywords:** dexamethasone, hepatocellular carcinoma, ischemia-reperfusion injury, liver resection, prognosis, randomized controlled trial

## Abstract

**Background:**

A previous randomized controlled trial demonstrated that intermittent Pringle’s maneuver (IPM) with a 25-min ischemic interval could be applied safely and efficiently in hepatectomy for patients with hepatocellular carcinoma (HCC). But prolonging the hepatic inflow clamping time will inevitably aggravate the ischemia-reperfusion injury. Therefore, we aimed to evaluate the effect of prophylactic dexamethasone on alleviating surgical stress for HCC patients with a 25-min ischemic interval.

**Methods:**

From December 2022 to April 2023, patients who met the inclusion criteria were randomly assigned to the dexamethasone group or control group. Perioperative data and short-term survival outcomes between the two groups were recorded and compared, and subgroup analysis was performed.

**Results:**

Two hundred and seventy patients were allocated to the dexamethasone group (*n*=135) and control group (*n*=135). Patients in the dexamethasone group had lower area under the curve of serial alanine aminotransferase (AUC_ALT_) (*P*=0.043) and aspartate aminotransferase (AUC_AST_) (*P*=0.009), total bilirubin (TB) (*P*=0.018), procalcitonin (PCT) (*P*=0.012), interleukin-6 (IL-6) (*P*=0.006), incidence of major complication (*P*=0.031) and shorter postoperative hospital stay (*P*=0.046) than those in the control group. Subgroup analysis showed that the dexamethasone group experienced milder hepatocellular injury than the control group for patients with cirrhosis, and for patients without cirrhosis, the dexamethasone group experienced milder inflammatory response. Moreover, the dexamethasone group preserved better liver function and experienced milder inflammatory response for patients undergoing major hepatectomy, although the hepatocellular injury was not significantly improved.

**Conclusion:**

Preoperative dexamethasone administration can help improve perioperative outcomes for HCC patients when applying IPM with a 25-min ischemic interval in hepatectomy.

## Introduction

Highlights

- When applying intermittent Pringle’s maneuver with a 25-min ischemic interval in hepatectomy, preoperative prophylactic dexamethasone can reduce surgical stress and confer benefits to the process of postoperative recovery.
- We explore the effect of dexamethasone on hepatocellular carcinoma (HCC) patients in subgroups with cirrhosis and after major hepatectomy. It could be brought into standard protocols for HCC patients undergoing liver resection.

Liver resection remains the most effective treatment for hepatocellular carcinoma (HCC). Meanwhile, controlling intraoperative blood loss remains a focus for surgeons^1^. Intermittent Pringle’s maneuver (IPM), with a cycle of 15-min inflow occlusion followed by 5-min reperfusion, is routinely used in many centers to prevent blood loss. However, one major concern of applying the IPM is ischemia-reperfusion injury (IRI) to the remnant liver^2,3^. Subsequently, a series of metabolic, immunological, microvascular, and inflammatory alterations will lead to cellular injury^4,5^.

Different hepatic clamping strategies have been reported to construct a state of protection against IRI, such as continuous Pringle maneuver or selective hepatic inflow occlusion^6,7^, prolonging the ischemic intervals during IPM^8–11^. One of them is our research, which demonstrates that IRI caused by 25-min IPM is not inferior to the 15-min IPM in HCC patients, and 25-min IPM is related to lower blood loss and higher speed for parenchyma transection. To balance the ischemia-induced hepatocellular damage and blood loss, we found that the optimal ischemic duration of a cycle during IPM is 25 min for our population.

Besides changing the clamping strategies, pharmacological interventions are equally indispensable. There are also randomized controlled trials (RCTs) examining perioperative steroid administration in liver resection. Positive effects on postoperative outcomes, such as improved liver function and lower incidence of postoperative complications, may be explained by the suppression of systemic inflammation and oxidative stress by reduction of inflammatory cytokines^12–18^.

Although the above studies have demonstrated that preoperative glucocorticoid administration may improve perioperative outcomes after liver resection, evidence supporting improved postoperative outcomes by prolonging the ischemic interval time in HCC patients is lacking. Therefore, we designed the trial to evaluate the effect of dexamethasone on postoperative short-term outcomes in HCC patients undergoing liver resection with 25-min IPM. Meanwhile, we explored the effect of dexamethasone on HCC patients with cirrhosis and after major hepatectomy.

## Methods

### Study design

The study was a randomized, dual-arm, parallel-group trial approved by the Biomedical Ethics Review Committee of our hospital and registered at the trial registry on 3 December 2022. This study was conducted in accordance with the principles of the Declaration of Helsinki and reported in line with Consolidated Standards of Reporting Trials (CONSORT) Guidelines^19^ (Supplemental Digital Content 1, http://links.lww.com/JS9/A827).

### Patients

All consecutive patients who received hepatic resection by four surgical groups in our department from 3 December 2022 to 16 April 2023 were screened. All patients who underwent liver resection for HCC were considered potential participants. Inclusion criteria were: (1) ischemic interval in IPM ≥25 min, (2) adequate functional reserve of important organ systems (heart, lungs, and kidneys), (3) normal liver function or well-reserved function before operation (Child–Pugh classes A or B with the score ≤7), (4) no other previous treatments before enrollment, such as portal vein embolization (PVE), transarterial chemoembolization (TACE), or systemic anti-tumor drug therapy. Exclusion criteria were: (1) aged <18 years or >80 years, (2) diseases receiving preceding systemic therapy with glucocorticoids, such as chronic kidney disease, inflammatory disease, or other immune system-related diseases, (3) anesthesiologist judge that the subjects cannot use the dexamethasone, such as diagnosed with epilepsy or active ulcer, (4) intraoperative findings of extrahepatic disease, need to undergo a synchronous resection for other organs except for the gallbladder, (5) intraoperative findings of additional intrahepatic lesions, need to combine with other procedures, such as ablation or biloenteric anastomosis, (6) unable to provide informed consents.

### Randomization and blinding

Patients were assigned randomly in a 1:1 ratio to the dexamethasone group or the control group on the day when they accepted surgery. The computer-generated numbers were prepared and kept inside sealed envelopes by a research assistant who was not physically present in the operating room. The block size was not disclosed to preserve allocation concealment.

When the patients entered the operating room, a research assistant opened the corresponding opaque envelope containing the patient’s allocation status and assigned medication. Then the research assistant referred the envelope to the anesthesiologist and reminded them to inject the medication before IPM. The medication container, appearance, dosage, and technique of administration were all the same for the two groups. Patients, surgeons, the nursing team, anesthesiologists, and outcome assessors were blinded to study allocation status. However, some cases could not be enrolled into the study due to unexpected intrahepatic or extrahepatic metastases that cannot be surgically removed, although comprehensive preoperative evaluations were performed.

### Operative procedure and invention

The surgical procedure was roughly identical to previous research^11^. A low central venous pressure anesthesia (below 5 cmH_2_O) was required for hepatectomy. The types of incision included inverse L-shape under the right costal margin or ‘laparoscopic five hole’. Intraoperative ultrasound was routinely performed to identify, count, and characterize the nature and vascular proximity of the tumor. IPM was performed at the hepatoduodenal ligament using a rubber sling for both open and laparoscopic hepatectomy. The liver parenchyma was dissected by harmonic scalpel (Ethicon Endo-Surgery, Cincinnati, Ohio), and the main hepatic vein or hepatic pedicle was transected by open or laparoscopic linear stapler (Ethicon Endo-Surgery, Cincinnati, Ohio). Hemostasis was achieved with dipolar coagulation (VIO 300 D system, ERBE, Germany), clips (Ethicon Endo-Surgery, LLC, Guaynabo, USA and/or titanium clips), or suturing for either open or laparoscopic hepatectomy. The major resection was defined as removing three or more segments^20^.

Patients in the dexamethasone group received intravenous administration of 10 mg dexamethasone (Dexamethasone Sodium Phosphate Injection, 1 ml:5 mg) 10 min before IPM. Patients in the control group received intravenous administration of 2 ml saline 10 min before IPM.

### Outcome measurement

All blood samples were collected on postoperative day (POD) 1, 2, 3, 4, 5, 6, and 7 following the same laboratory testing procedure. The primary outcomes were the transaminase-based postoperative hepatocellular injury indexes, including consecutive 7-day transaminase levels of alanine aminotransferase (ALT) and aspartate aminotransferase (AST) and their peak values. Besides, the area under the curve (AUC) of the postoperative course of ALT and AST was assessed.

The secondary outcomes included intraoperative blood loss, liver function parameters [total bilirubin (TB), international normalized ratio (INR), and their respective peaks], inflammatory cytokines, postoperative complications, postoperative hospital stay, and 30-day mortality. The intraoperative blood loss was assessed by the extraction of blood in the suction apparatus, subtracting the amount of saline used for intraoperative irrigation plus the assessment amount of blood in the gauze roll (by weighting the soaked gauzes). Inflammatory cytokines level was assessed by interleukin-6 (IL-6), procalcitonin (PCT), C-reactive protein (CRP), and their respective peaks. Complications were classified and recorded according to the Clavien–Dindo grade^21^. Liver failure was defined according to the ‘50–50 criteria’ on POD 5^22^; Hemorrhage was defined as a postoperative hemoglobin level dropping more than 3 g/dl compared to the postoperative baseline level^23^; Bile leak was determined by an increase in bilirubin concentration in abdominal drainage of more than three times compared to that in serum on POD 3^24^; Ascites was identified according to postoperative daily drainage fluid levels greater than 10 ml/kg of body weight^25^. The fibrosis stage was judged by the Ishak scoring system.

### Sample size

This trial was originally conceived by the statistical and clinical teams with a total of 270 patients (135 within each group). It was based on our previous RCT^11^ and the other relative RCT in our center^7^. In our previous study, the standard deviation (σ) of the postoperative serum peak ALT values was 184.684 IU/l between the two groups and the mean difference (δ) was 58.882 IU/l. Thus, we estimated a sample size of 135 patients in each group to achieve an 80% statistical power (Uβ=0.84, one-sided test) at the 5% significance level (Uα=1.645, one-sided test), as well as an estimated dropout rate of ~10%.

### Statistical analysis

Continuous variables were presented as median (range) and compared by the Mann–Whitney *U* test. Categorical variables were presented as numbers (percentage) and compared using the chi-square (*χ* ^2^) test or Fisher’s exact test. The modified intention-to-treat principled analysis was applied to allocate patients to the original group. Sensitivity analysis of missing data was conducted with the multiple-imputation approach. All tests for differences were two-tailed and considered statistically significant if *P* values <0.05. All statistical analyses were performed by the SPSS version 20 and GraphPad Prism version 8.0.1.

The significance of postoperative complications was analyzed using *χ* ^2^ test or Fisher’s exact test, tabulated and summarized by using descriptive statistics.

Besides, according to the background of hepatic fibrosis and the extent of resection, subgroup analyses were performed to assess the protective effect of the administration of dexamethasone before clamping the hilar pedicle on residual liver injury and inflammatory response after liver resection.

## Results

### Patients

**Figure 1:**
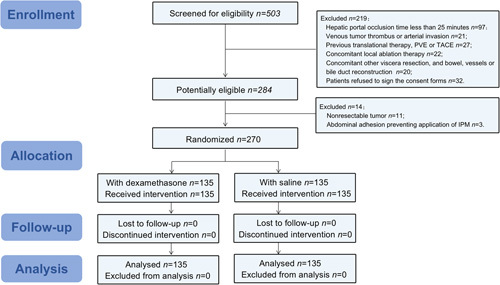
Flowchart of study participants. IPM, intermittent Pringle maneuver; PVE, portal vein embolization; TACE, transarterial chemoembolization.

A total of 503 consecutive patients undergoing hepatectomy for HCC were screened for this trial. Two hundred and thirty-three patients were excluded from this study for the following reasons: total hepatic portal occlusion time was less than 25 min for 97; venous tumor thrombus or arterial invasion for 21; previous translational therapy, PVE or TACE for 27; concomitant local ablation therapy for 22, concomitant other viscera resection, and bowel, vessels or bile duct reconstruction for 20. In addition, 32 patients refused to sign the consent forms. Another 14 patients had nonresectable tumor or abdominal adhesion, preventing the application of IPM after exploration. Finally, out of these 270 patients, 135 patients were assigned to the dexamethasone group and 135 patients to the control group, as shown in Figure 1.

### Baseline data

Table 1 displayed the patients’ baseline characteristics and preoperative laboratory examinations, including age, gender, body mass index (BMI), serum TB, ALT, AST, creatinine, albumin, INR, platelet, CRP, IL-6, PCT, and the percentage of serum positive hepatitis B surface antigen, hepatitis B virus deoxyribonucleic acid (HBV DNA) ≥1000 IU/ml, alpha-fetoprotein (AFP) ≥400 ng/ml, Child–Pugh grade A, indocyanine green retention rate at 15 min (ICG-R15) ≤10%, and portal hypertension. There were no significant differences between the two groups.

**Table 1:** Baseline characteristics of HCC patients undergoing hepatectomy.

	Dexamethasone group (*n*=135)	Control group (*n*=135)	*P*
Demographics			
Age, year, median (range)	54 (22–80)	56 (24–79)	0.780
Male, *n* (%)	120 (88.9)	117 (86.7)	0.577
BMI, kg/m^2^, median (range)	23.66 (16.80–29.76)	23.53 (17.99–33.53)	0.663
Preoperative lab tests			
HBsAg positivity, *n* (%)	121 (89.6)	113 (83.7)	0.152
HBV DNA ≥1000 IU/ml, *n* (%)	37 (27.4)	36 (26.7)	0.891
AFP ≥400 ng/ml, *n* (%)	34 (25.2)	40 (29.6)	0.413
TB level, μmol/l, median (range)	13.20 (4.00–32.80)	12.70 (3.60–31.40)	0.979
ALT level, IU/l, median (range)	31 (8–160)	31 (6–168)	0.814
AST level, IU/l, median (range)	29 (2–145)	30 (13–163)	0.955
Creatinine, μmol/l, median (range)	80 (46–179)	78 (42–218)	0.424
Alb level, g/l, median (range)	42.6 (33.9–52.7)	43.3 (31.2–52.5)	0.355
INR, median (range)	1.04 (0.80–1.39)	1.02 (0.84–1.31)	0.309
Platelet count, 10^9^/l, median (range)	124 (34–328)	134 (44–410)	0.199
CRP level, mg/l, median (range)	3.91 (0.86–88.33)	3.91 (0.37–97.10)	0.413
IL-6 level, pg/ml, median (range)	4.97 (1.21–87.90)	4.99 (0.85–77.74)	0.657
PCT level, ng/ml, median (range)	0.035 (0.01–0.55)	0.036 (0.01–0.53)	0.556
Child–Pugh grade A, *n* (%)	128 (94.8)	126 (93.3)	1.000
ICG-R15 ≤10%, *n* (%)	129 (95.6)	124 (91.9)	0.210
PHT, *n* (%)	42 (31.1)	35 (25.9)	0.345

AFP, alpha-fetoprotein; Alb, albumin; ALT, alanine aminotransferase; AST, aspartate aminotransferase; BMI, body mass index; CRP, C-reactive protein; HBsAg, hepatitis B surface antigen; HBV DNA, hepatitis B virus deoxyribonucleic acid; HCC, hepatocellular carcinoma; ICG-R15, indocyanine green retention rate at 15 min; IL-6, interleukin-6; INR, international normalized ratio; PCT, procalcitonin; PHT, portal hypertension; TB, total bilirubin.

### Intraoperative characteristics

As shown in Table 2, the total operation time, number of cycle of IPM, total ischemic time, total release time, and transection area were comparable between the two groups. Moreover, the intraoperative blood loss and the rate of blood transfusion did not differ in the two groups, although we delicately divided the blood loss into four sections (<100 ml, 100–500 ml, 500–1000 ml, ≥1000 ml). Besides, the types of hepatectomy and the rate of laparoscopic hepatectomy were comparable between the two groups; the proportion of major hepatectomy was 33.3% in the dexamethasone group and 29.6% in the control group, respectively (*P*=0.512).

**Table 2:** Intraoperative parameters and postoperative outcome measures of HCC patients undergoing hepatectomy.

	Dexamethasone group (*n*=135)	Control group (*n*=135)	*P*
Intraoperative parameters			
Total operation time, min, median (range)	192 (85–373)	200 (90–470)	0.271
No. of the cycle of IPM, median (range)	2 (1–5)	2 (1–6)	0.333
Transection time, min, median (range)	49 (25–142)	46 (25–155)	0.151
Total ischemic time, min, median (range)	45 (25–122)	41 (25–130)	0.116
Total release time, min, median (range)	5 (0–20)	5 (0–25)	0.333
Transection area, cm^2^, median (range)	65.5 (10.5–235.3)	55.8 (12.8–244.3)	0.438
Total blood loss, ml, median (range)	249.0 (20.0–2756.0)	215.0 (30.0–2331.0)	0.286
<100 ml, *n* (%)	11 (8.1)	17 (12.6)	0.231
100–500 ml, *n* (%)	106 (78.5)	99 (73.3)	0.319
500–1000 ml, *n* (%)	14 (10.4)	17 (12.6)	0.567
≥1000 ml, *n* (%)	4 (3.0)	2 (1.5)	0.684
No. of blood transfusion, *n* (%)	16 (11.9)	15 (11.1)	0.849
Open/laparoscopic hepatectomy, *n*/*n*	97/38	86/49	0.152
Type of resection			
Wedge resection(s)	29 (21.5)	30 (22.2)	0.883
Left lateral sectionectomy	4 (3.0)	3 (2.2)	1.000
Right posterior sectionectomy	25 (18.5)	29 (21.5)	0.543
Segmentectomy	11 (8.1)	14 (10.4)	0.529
Bisegmentectomy	21 (15.6)	19 (14.1)	0.732
Trisectionectomy	12 (8.9)	11 (8.1)	0.827
Left hemi-hepatectomy	14 (10.4)	11 (8.1)	0.529
Right hemi-hepatectomy	16 (11.9)	14 (10.4)	0.699
Extended left/right hepatectomy	3 (2.2)	4 (3.0)	1.000
Minor/major hepatectomy, *n*/*n*	90/45	95/40	0.512
Postoperative lab tests			
Peak ALT, IU/l, median (range)	293 (35–1652)	338 (75–2156)	0.114
AUC_ALT_, U/(l×day), median (range)	1104 (215–5799)	1128 (375–6194)	0.043
Peak AST, IU/l, median (range)	271 (48–987)	304 (80–1128)	0.095
AUC_AST_, U/(l×day), median (range)	710 (199–3024)	804 (305–2326)	0.009
Peak TB, μmol/l, median (range)	35.1 (16.1–151.5)	38.7 (18.8–108.6)	0.018
Peak INR, median (range)	1.34 (1.03–2.12)	1.32 (1.02–2.0)	0.537
Peak PCT, ng/ml, median (range)	1.06 (0.31–5.12)	1.15 (0.49–6.58)	0.012
Peak IL-6, pg/ml, median (range)	165 (61.2–760)	193 (64.5–878)	0.006
Peak CRP, mg/l, median (range)	99.5 (40.5–320)	105 (40–304)	0.093
Pathology of tumor			
Single tumor, *n* (%)	115 (85.2)	106 (78.5)	0.155
Largest tumor size, cm, median (range)	3.7 (1.0–17.0)	4.0 (0.8–15.0)	0.214
Resected margin positivity, *n* (%)	0 (0)	0 (0)	–
Microvascular invasion, *n* (%)	57 (42.2)	65 (48.1)	0.328
Tumor grade, *n* (%)			
G1	6 (4.4)	3 (2.2)	0.500
G2	90 (66.7)	86 (63.7)	0.609
G3	39 (28.9)	46 (34.1)	0.359
Pathology of non-tumorous liver			
Presence of fibrosis, *n* (%)	129 (95.6)	128 (94.8)	0.776
Fibrosis stage			
Early, (Ishak 1–2), *n* (%)	6 (4.4)	4 (3.0)	0.519
Intermediate, (Ishak 3–4), * n* (%)	67 (49.6)	66 (48.9)	0.903
Advanced; cirrhosis, (Ishak 5–6), *n* (%)	56 (41.5)	58 (43.0)	0.805
Complications	51 (37.8)	64 (47.4)	0.110
Grade I–II	32 (23.7)	31 (23.0)	0.886
Wound infection	5 (3.7)	3 (2.2)	0.722
Abdominal abscess	2 (1.5)	0 (0)	0.498
Hematologic disorder	4 (3.0)	3 (2.2)	1.000
Cardiac arrhythmia	2 (1.5)	3 (2.2)	1.000
Electrolyte disorders	5 (3.7)	6 (4.4)	0.758
Intestinal obstruction	2 (1.5)	3 (2.2)	1.000
Pulmonary infection	8 (5.9)	9 (6.7)	0.802
Urinary tract infection	2 (1.5)	3 (2.2)	1.000
Deep vein thrombosis	2 (1.5)	1 (0.7)	1.000
Grade III–V	19 (14.1)	33 (24.2)	0.031
Ascites	3 (2.2)	7 (5.2)	0.197
Wound bleeding	1 (0.7)	1 (0.7)	1.000
Abdominal abscess	1 (0.7)	2 (1.5)	1.000
Bile leakage	4 (3.0)	5 (3.7)	1.000
Abdominal bleeding	2 (1.5)	1 (0.7)	1.000
Intestinal obstruction	1 (0.7)	3 (2.2)	0.622
Pleural effusion	5 (3.7)	7 (5.2)	0.555
Liver failure	2 (1.5)	6 (4.4)	0.282
Renal failure	0 (0)	1 (0.7)	1.000
Death	0 (0)	0 (0)	–
No. of entering into ICU, *n* (%)	3 (2.2)	4 (3.0)	1.000
Postoperative hospital stay, day, median (range)	6 (5–16)	7 (5–15)	0.046
30-day mortality, *n* (%)	0 (0)	1 (0.7)	1.000

ALT, alanine aminotransferase; AST, aspartate aminotransferase; AUC_ALT_, area under the curve of alanine aminotransferase; AUC_AST_, area under the curve of aspartate aminotransferase; CRP, C-reactive protein; HCC, hepatocellular carcinoma; ICU, intensive care unit; IL-6, interleukin-6; INR, international normalized ratio; IPM, intermittent Pringle maneuver; PCT, procalcitonin; TB, total bilirubin.

### Postoperative relevant parameters

**Figure 2:**
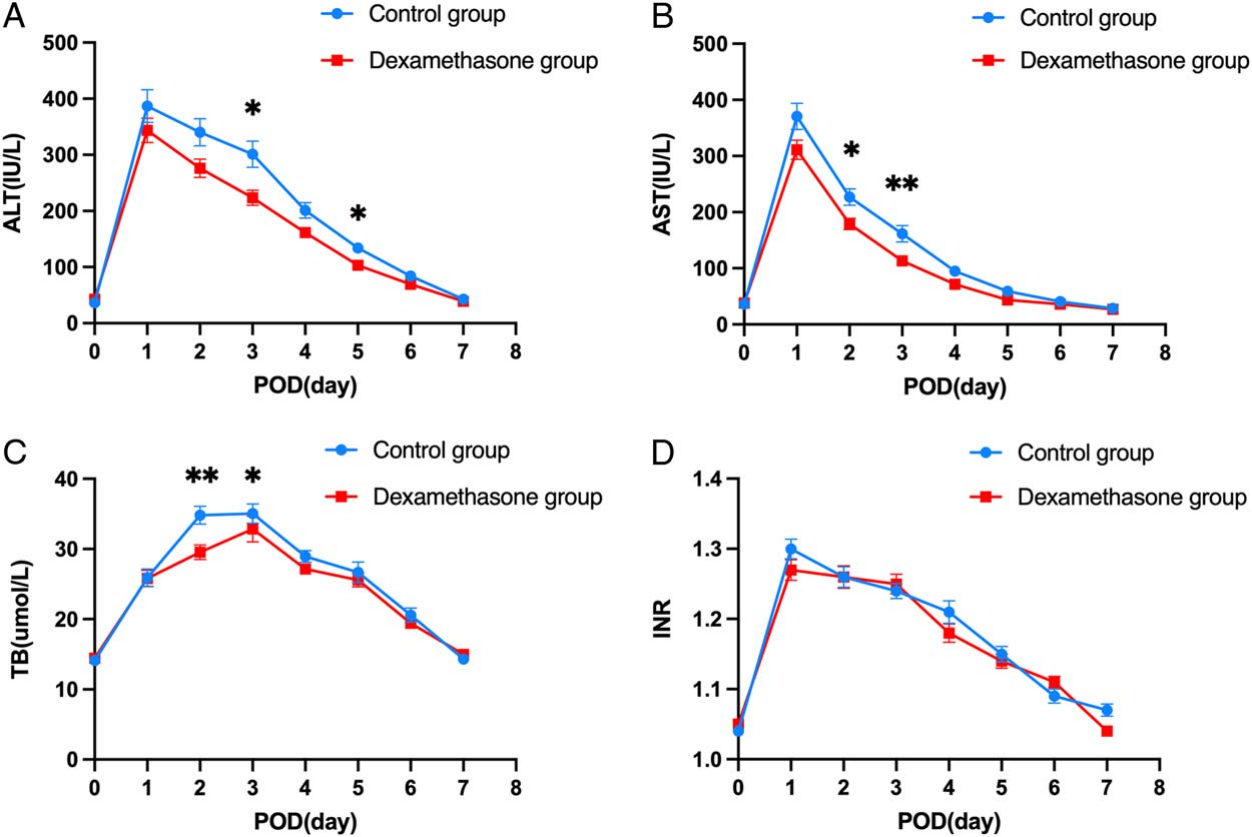
Serial measurements of serum (A) ALT, (B) AST, (C) TB, and (D) INR for the dexamethasone and control group. Data were presented as mean±standard error. ALT, alanine aminotransferase; AST, aspartate aminotransferase; INR, international normalized ratio; POD, postoperative day; TB, total bilirubin. (*P<0.05; **P<0.01).

**Figure 3:**
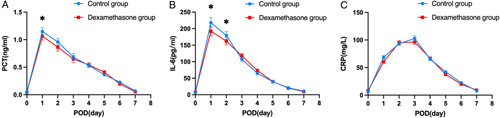
Serial measurements of serum (A) CRP, (B) IL-6, and (C) PCT for the dexamethasone and control group. Data are presented as mean±standard error. CRP, C-reactive protein; IL-6, interleukin-6; PCT, procalcitonin; POD, postoperative day. (*P<0.05).

In Figure 2A–D and Figure 3A–C, we depicted the serial 8-day changes in ALT, AST, TB, INR, PCT, IL-6, and CRP from preoperative to POD 7, respectively. The ALT, AST, INR, PCT, and IL-6 levels reached to peak on POD 1, CRP levels reached to peak on POD 3, and all of them returned to preoperative values within 7 days for most patients. The TB level reached to peak in POD 2 for the control group but on POD 3 for the dexamethasone group for most patients. There were some scattered differences of ALT, AST, TB, PCT, and IL-6 levels emerging in the postoperative course.

**Figure 4:**
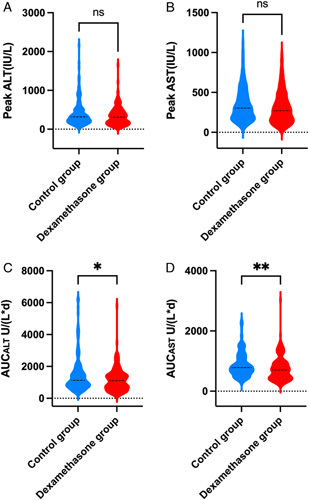
Violin plots showing the (A) peak ALT, (B) peak AST, (C) AUC_ALT_ and (D) AUC_AST_ levels for the dexamethasone and control group. Solid lines represented the median value. ALT, alanine aminotransferase; AST, aspartate aminotransferase; AUC_ALT_, area under the curve of alanine aminotransferase; AUC_AST_, area under the curve of aspartate aminotransferase. (*P<0.05; **P<0.01; ns, no significance)

As shown in Table 2 and Fig. 4A–D, there was a tendency for operative median peak ALT and AST to be relatively lower in the dexamethasone group than in the control group (*P*=0.114 and *P*=0.095, respectively), even though the difference were not statistically significant. However, when the median AUC was compared, the dexamethasone group was associated with smaller AUC_ALT_ and AUC_AST_ values compared with the control group (*P*=0.043 and *P*=0.009, respectively).

When liver function was assessed by serum TB and INR, the median peak TB level was significantly lower in the dexamethasone group than in the control group (*P*=0.018), but no difference was in median peak INR between the two groups (Table 2). Otherwise, there were differences in median peak PCT, IL-6, and CRP; the dexamethasone group was associated with lower inflammatory cytokines levels compared with the control group, especially in PCT and IL-6 (*P*=0.012 and *P*=0.006, respectively) (Table 2).

Table 2 showed that there were no significant differences in the tumor pathological characteristic between the two groups, including the percentage of single tumors, positive resected margin, microvascular invasion, tumor grade, and the largest tumor size. For para-cancerous liver, 129 patients (95.6%) had fibrosis in the dexamethasone group, of whom 6 (4.4%) had early fibrosis, 67 (49.6%) had intermediate fibrosis, and 56 (41.5%) had cirrhosis. Similarly, 128 patients (94.8%) had fibrosis in the control group, of whom 4 (3.0%) had early fibrosis, 66 (48.9%) had intermediate fibrosis, and 58 (43.0%) had cirrhosis. Both proportions of fibrosis and fibrosis stages were comparable between the two groups.

### Short-term prognosis

As shown in Table 2, the overall complication rate of the dexamethasone group and control group was 37.8% and 47.4%, respectively (*P*=0.110). However, there was a significant difference between the two groups regarding major (grade III–IV) complications, 19 (14.1%) in the dexamethasone group and 33 (24.2%) in the control group (*P*=0.031), and patients had a longer postoperative hospital stay in the control group than in dexamethasone group (*P*=0.046). In the control group, a 56-year-old patient died of hypertensive cerebral hemorrhage within 30 days after surgery. Moreover, no significant difference was found between both groups in the number of entering the intensive care unit (ICU) after surgery.

### Subgroup analysis

As shown in Table 3 and Supplementary Figure 1A–L (Supplemental Digital Content 2, http://links.lww.com/JS9/A828), for the subgroup of patients with cirrhosis, patients in the dexamethasone group had significantly lower levels of AUC_ALT_, AUC_AST_, and peak TB than those in the control group (*P*=0.022, *P*=0.001, and *P*=0.009, respectively), although the peak ALT and AST were statistically comparable between the two groups. Similarly, no significant differences were observed in INR, CRP, IL-6, and PCT levels between the two groups. However, for patients without cirrhosis, patients in dexamethasone group had lower levels of CRP, IL-6, and PCT (*P*=0.068, *P*=0.040, and *P*=0.021, respectively), but the hepatocellular injury indexes and liver function parameters showed no significant difference between the two groups (peak ALT: *P*=0.605, AUC_ALT_: *P*=0.543; peak AST: *P*=0.216, AUC_AST_: *P*=0.092; peak TB: *P*=0.105, peak INR: *P*=0.472).

**Table 3:** Subgroup analysis according to cirrhosis.

	Cirrhosis			Without cirrhosis		
Parameters	Dexamethasone group (*n*=56)	Control group (*n*=58)	*P*	Dexamethasone group (*n*=79)	Control group (*n*=77)	*P*
Peak ALT, IU/l, median (range)	303 (52–1652)	339 (75–1708)	0.162	293 (35–973)	299.5 (84–2156)	0.605
AUC_ALT_, U/(l×day), median (range)	1158.5 (220–5799)	1251.5 (397–6194)	0.022	1030 (215–2779)	1087 (375–5711)	0.543
Peak AST, IU/l, median (range)	281 (48–987)	348.5 (103–1128)	0.056	240 (53–895)	290 (80–987)	0.216
AUC_AST_, U/(l×day), median (range)	752 (199–3024)	850.5 (471.5–2249)	0.001	699 (218–1858)	736 (305–2326)	0.092
Peak TB, μmol/l, median (range)	36.95 (16.40–126.1)	41.85 (20.90–93.3)	0.009	34.9 (16.1–151.5)	37.2 (18.8–108.6)	0.105
Peak INR, median (range)	1.36 (1.03–2.12)	1.38 (1.1–2.0)	0.662	1.26 (1.08–2.0)	1.31 (1.02–1.68)	0.472
Peak CRP, mg/l, median (range)	108.2 (40.5–241)	110.6 (40–304)	0.934	99.5 (40.8–320)	113 (56–298)	0.068
Peak IL-6, pg/ml, median (range)	161 (61.2–757)	187 (64.5–878)	0.092	170 (68.4–760)	201 (82.2–824)	0.040
Peak PCT, ng/ml, median (range)	1.09 (0.32–4.31)	1.16 (0.49–5.16)	0.263	1.12 (0.31–5.12)	1.25 (0.56–6.58)	0.021

ALT, alanine aminotransferase; AST, aspartate aminotransferase; AUC_ALT_, area under the curve of alanine aminotransferase; AUC_AST_, area under the curve of aspartate aminotransferase; CRP, C-reactive protein; IL-6, interleukin-6; INR, international normalized ratio; PCT, procalcitonin; TB, total bilirubin.

As shown in Table 4 and Supplementary Figure 2A–L (Supplemental Digital Content 2, http://links.lww.com/JS9/A828), for the subgroup of patients who underwent major hepatectomy, the hepatocellular injury indexes (peak ALT and AST, AUC_ALT_, and AUC_AST_) showed no significant difference between dexamethasone group and control group (*P*=0.495, *P*=0.374, *P*=0.134, and *P*=0.481 respectively). The liver function parameters and inflammatory cytokines levels were significantly lower in the dexamethasone group (peak TB: *P*=0.034, peak INR: *P*=0.032, peak CRP: *P*=0.048, peak IL-6: *P*=0.005, peak PCT: *P*=0.012). Besides, for patients who underwent minor hepatectomy, we observed that patients in the dexamethasone group had lower levels of AUC_AST_ and peak TB (*P*=0.010 and *P*=0.048, respectively), and the other hepatocellular injury indexes and liver function parameters, inflammatory cytokines levels, showed no significant difference between the two groups.

**Table 4:** Subgroup analysis according to the extent of resection.

	Major hepatectomy			Minor hepatectomy		
Parameters	Dexamethasone group (*n*=45)	Control group (*n*=40)	*P*	Dexamethasone group (*n*=90)	Control group (*n*=95)	*P*
Peak ALT, IU/l, median (range)	317 (45–780)	326 (85–1585)	0.495	290 (35–1652)	313 (75–2156)	0.398
AUC_ALT_, U/(l×day), median (range)	1147 (215–2779)	1309.5 (416–4272)	0.134	1054 (281–5799)	1087 (375–6194)	0.149
Peak AST, IU/l, median (range)	309 (48–896)	324 (117–1128)	0.374	262 (53–987)	291 (80–987)	0.171
AUC_AST_, U/(l×day), median (range)	774.5 (199–1858)	813.5 (401–2249)	0.481	687.75 (218–3024)	799.5 (305–2326)	0.010
Peak TB, μmol/l, median (range)	38.5 (20.4–143.6)	42.15 (22.4–93.3)	0.034	33.8 (16.1–151.5)	38.3 (18.8–108.6)	0.048
Peak INR, median (range)	1.33 (1.03–1.84)	1.41 (1.1–2.0)	0.032	1.26 (1.08–2.12)	1.31 (1.02–1.78)	0.164
Peak CRP, mg/l, median (range)	99.5 (40.5–272)	112.05 (40–298)	0.048	102 (40.8–320)	111 (49.4–304)	0.098
Peak IL-6, pg/ml, median (range)	166 (74.3–643)	221 (97.1–878)	0.005	163 (61.2–760)	189 (64.5–781)	0.147
Peak PCT, ng/ml, median (range)	1.1 (0.31–4.94)	1.195 (0.55–6.58)	0.012	1.08 (0.33–5.12)	1.15 (0.49–3.38)	0.210

ALT, alanine aminotransferase; AST, aspartate aminotransferase; AUC_ALT_, area under the curve of alanine aminotransferase; AUC_AST_, area under the curve of aspartate aminotransferase; CRP, C-reactive protein; IL-6, interleukin-6; INR, international normalized ratio; PCT, procalcitonin; TB, total bilirubin.

## Discussion

The development process of liver surgery is accompanied by a history of struggling with bleeding. Because blood loss and red blood cell transfusion promote HCC recurrence and reduce long-term survival after radical resection^1,26^. Although various liver transection instruments and hemostatic techniques have been continuously updated and developed, IPM is still widely applied in hepatectomy as its ease and effectiveness in controlling bleeding, as well as the rapid development of laparoscopic liver resection^27^. However, the main controversy of the IPM is how to compromise the contradiction between IRI and blood loss. Our previous RCT showed that prolonging the IPM time to 25 min had less blood loss compared with the standard 15 min; meanwhile, the residual liver injury was not significantly aggravated. In the past two decades, we consulted that there are eight RCTs referring to the impact of perioperative steroids administration in liver resection^12–15,17,18,28,29^. But limitations of these reports included, but are not restricted to, small sample size, different underlying liver disorders and extent of liver resection, inconsistent glucocorticoid preparation, timing of administration and dosage, and a discrepancy of surgical indications. Given these challenges, our group elected to conduct a RCT to validate the effect of dexamethasone administration on alleviating surgical stress in hepatectomy with 25-min ischemic interval in IPM, for a specific population diagnosed with HCC.

Compared with liver benign tumors or liver metastasis, patients with HCC in our country were characterized by cirrhosis, portal hypertension, and dysfunction of the coagulation mechanism, which put tremendous surgical pressure on the residual liver. As a result, the residual liver will undergo a series of pathophysiological alterations, such as producing inflammatory protein, modifying the hemostatic balance, and altering metabolism^12,30^. Among these, inflammatory cytokines played a prominent role. Muratore *et al*.^15^ demonstrated that IL-6 was related to the degree of tissue damage and systemic inflammatory response and was a reliable marker of the magnitude of surgical trauma. Most studies evaluating this topic have identified a temporization of rising postoperative inflammatory cytokines levels with glucocorticoid use^12,14,15,17,18^. Our study confirmed a similar trend of inflammatory cytokines in consecutive 7 days after surgery. This observation significantly extended to both peak levels of IL-6 and PCT.

The degree of IRI induced by IPM was assessed by postoperative consecutive 7 days serum ALT and AST levels and their respective peak in our study. The transaminase peak occurred on POD 1 for almost all patients. Our data showed that the dexamethasone group possessed a smoother upward trend and faster downward trend of ALT and AST levels in the postoperative course. Peak ALT and AST had no significant difference compared with the control group, and this point was not identical to three RCTs in Western countries ^12,15,17^. A well-designed study with a sufficient sample size conducted in Japan showed the same results as ours that there were no significant differences between the groups of ALT or AST levels every day after surgery, as well as their peaks^14^. We speculated that the principal reason was the different composition ratios of patient cohorts. Colorectal liver metastases dominated the western study, while our and Japanese studies mainly focused on HCC. Therefore, we considered that chemotherapy-related liver parenchyma had a different response to steroids than hepatitis-related liver. To accurately evaluate the transaminase-based postoperative hepatic injury, we calculated the AUC formed by postoperative serum aminotransferase levels and found that the AUC_ALT_ and AUC_AST_ were significantly smaller in the dexamethasone group. Compared to daily or peak transaminase values, the AUC comprehensively reflected the overall course of postoperative hepatocellular damage^8^.

Bilirubin, as an indispensable and reliable indicator for judging postoperative liver failure, was highly valued by clinicians^31^. Hayashi’s trial chose the TB as the primary outcome^14^, drawing a conclusion that there were significant differences in bilirubin level during the 7-day course after an operation, especially in POD 2 between the experiment group and control group. They administrated the experimental group with a preoperative dose of 500 mg hydrocortisone, then 300 mg on POD 1, 200 mg on POD 2, and 100 mg on POD 3. This high-dose glucocorticoid administration was not routinely applicable to HCC patients. Compared with our prophylactic dose of dexamethasone, it also had similar effects on delaying the arrival of TB peak and lowering overall levels. Although bilirubin was a reliable indicator, considering that only 3 of 105 patients had a bilirubin value >3 mg/ml after liver resection in Hayashi’s study, we abandoned bilirubin as a primary outcome.

As far as we know, the postoperative potential effects of glucocorticoid in liver cirrhosis and major hepatectomy patients were not elucidated. In the subgroup with cirrhosis, the dexamethasone group had smaller AUC_ALT_, AUC_AST_, and lower TB levels, which implied that the protective effects of dexamethasone were more pronounced in an unsatisfactory liver background. Inflammatory cytokines were important indexes reflecting hepatic IRI. In addition, intestinal injury after IPM might result in intestinal barrier dysfunction and bacterial translocation^32,33^. Therefore, the inflammatory cytokines plasma concentration can also be regarded as an index reflecting intestinal injury secondary to portovenous stasis^34,35^. Our study showed that dexamethasone significantly reduced the inflammatory response, reflected by the peak levels of inflammatory cytokines, such as IL-6 and PCT. However, an unanticipated finding in our stratification analysis was that the differences in IL-6 and PCT between the dexamethasone group and control group disappeared in patients with cirrhosis. This might be explained that the presence of collateral circulation and the absence of portal congestion in patients with a cirrhotic liver made them better tolerant to portal cross-clamping. Major hepatectomy was closely monitored because of a more pronounced inflammatory response and a higher risk of liver failure and death. Bressan *et al*.^36^ designed a RCT of the effects of preoperative single-dose methylprednisolone after major liver resection. They concluded that administration of preoperative single-dose methylprednisolone in patients undergoing a subsequent major liver resection was associated with better short-term outcomes. However, they were unable to track postoperative inflammatory cytokines levels. In our study, not only did it confirm that preoperative glucocorticoid administration contributed to reducing complications and improving survival outcomes, but it also demonstrated the important role of glucocorticoids in relieving surgical stress and suppressing the inflammatory response.

The overall complication rate was not significantly different between the two groups (37.8 vs. 47.4%). However, the major complication rate was noted to be significantly lower in the dexamethasone group (14.1 vs. 24.2%, *P*=0.031). This was mainly influenced by a higher rate of ascites and liver failure in the control group (9.6 vs. 3.7%). This observation fits quite well with previous studies that identified the relationship between steroids and complications after hepatectomy^13,16^. In Yamashita’s, Muratore’s, and Hayashi’s studies, they did not report a difference in postoperative overall or major complication rates^14,15,30^, we observed that the minor hepatectomy was close to 90% in their cohorts. In our study, not only did it exclude patients with ischemia time less than 25 min, but also the major hepatectomy was more than 30%. Raised levels of IL-6 have consistently been reported to correlate with postoperative complications^37,38^. Similarly, our study confirmed an increased rate of major complications in patients with higher postoperative IL-6 levels. With the reduction of major complications, the length of stay was also notably shorter in the dexamethasone group. Of course, this desired outcome also benefitted from the effects of dexamethasone, including analgesia, antiemesis, and anti-inflammatory^39^.

Although the preoperative administration of dexamethasone seemed to be beneficial for patients’ recovery, there were some concerns about negative effects on wound healing and infection^40,41^. Moreover, there are possible bleeding complications due to gastrointestinal ulcers and a potential risk of thromboembolic events. In our study, gastrointestinal ulcer bleeding and thromboembolic events were rare, which might be attributed to the prophylactic administration of proton pump inhibitors and low molecular weight heparin in high-risk patients. Infection events were not different between groups in our study. Not only that, Pulitano and Bressan reported that preoperative steroid administration could reduce the related infection events^17,36^.

Limitations of the present study must be considered. Firstly, choosing dexamethasone as preoperative steroids administration had no credible evidence. Because there is currently no international organization to make recommendations to standardize the perioperative use of steroids in patients undergoing hepatectomy. But compared with other studies using high-dose glucocorticoids, our research had important exploratory significance and also yielded clinically significant results. Second, we did not consider the impacts of hepatic vascular anomaly on hepatic blood flow clamping, which may cause bias in postoperative outcomes. Third, this study was a single-center trial focusing on most hepatitis B virus (HBV)-related HCC, which might not be generalizable to other centers or populations. Fourth, it was designed to look only at the postoperative short-term outcomes; long-term oncologic effects of the dexamethasone need further exploration. Finally, magnesium isoglycyrrhizinate, which has anti-inflammatory, protective effects on hepatocyte membrane and improves liver function, is usually administrated for patients after hepatectomy in our center. Therefore, the effects of dexamethasone alone are somewhat hard to evaluate based on the current protocol, but all the patients received magnesium isoglycyrrhizinate treatment from the first day after surgery.

## Conclusion

In summary, this prospective randomized study demonstrated that preoperative glucocorticoid management is a safe and effective modulator of surgical stress. Preoperative prophylactic dexamethasone can reduce IRI and confer benefits to the process of postoperative liver recovery. Meanwhile, it can reduce postoperative complications and shorten postoperative hospital stays. We propose that preoperative dexamethasone administration should be brought into standard protocols for HCC patients undergoing liver resection.

## Ethical approval

The study was approved by the Biomedical Ethics Review Committee of West China Hospital of Sichuan University (Approval number: 2022-1757; Date: 2022-11-28).

## Consent

Written informed consent was obtained from the patient for publication and any accompanying images. A copy of the written consent is available for review by the Editor-in-Chief of this journal on request.

## Financial support and sponsorship

This study was supported by China Postdoctoral Science Foundation, Grant/Award Number: 2022M712263; Key Technology Research and Development Program of the Sichuan Province, Grant/Award Number: 2021YFSY0009 and 2021YFS0106; National Natural Science Foundation of China, Grant/Award Number: 82203785 and 82270648; Post-Doctor Research Project, West China Hospital, Sichuan University, Grant/Award Number: 2020HXBH076 and Key Clinical Research Incubation Project of West China Hospital of Sichuan University, Grant/Award Number: 2020HXFH028.

## Author contribution

Y.H. and L.J.: conceptualization; Y.H. and N.W.: data curation; L.X.: formal analysis; L.X. investigation; Y.H. and L.X.: methodology; L.J. and M.X.: supervision; Y.W. and W.W.: validation; Y.H.: writing – original draft; L.J. and M.X.: writing – review and editing.

## Conflicts of interest disclosure

The authors report no conflicts of interest in this work.

## Research registration unique identifying number (UIN)

1. Name of the registry: Chinese Clinical Trial Registry chictr.org.cn
2. Unique identifying number or registration ID: ChiCTR220006638.
3. Hyperlink to your specific registration (must be publicly accessible and will be checked): https://www.chictr.org.cn/showproj.html?proj=187146.

## Guarantor

Li Jiang and Mingqing Xu.

## Data availability statement

All detailed data included in the study are available upon request by contact with the corresponding author.

## Provenance and peer review

Not commissioned, externally peer-reviewed.

## Presentation

None.

## Supplementary Material
