# Supplementary material for: Preoperative dexamethasone administration in hepatectomy of 25-min intermittent Pringle’s maneuver for hepatocellular carcinoma: a randomized controlled trial
Source: Int J Surg. 2023 Jul 31;109(11):3354–64. doi: 10.1097/JS9.0000000000000622 (PMC10651268; doi:10.1097/JS9.0000000000000622)
Supplement: Supplementary file 2 [file js9-109-3354-s002.pdf]

Supplementary figures

Supplementary Figure 1: Serial measurements of serum ALT, AST in subgroup of cirrhosis (A-B) and without cirrhosis (C-D) for the dexamethasone and control group. Data were presented as mean ± standard error. Violin plots showing the peak ALT, peak AST, AUC_{ALT} and AUC_{AST} levels in subgroup of cirrhosis (E-H, respectively) and without cirrhosis (I-L, respectively) for the dexamethasone and control group. Solid lines represented median value. ALT, alanine aminotransferase; AST, aspartate aminotransferase; AUC, area under of the curve; POD, postoperative day. (*: $p<0.05$; **: $p<0.0$; ns: no significance)

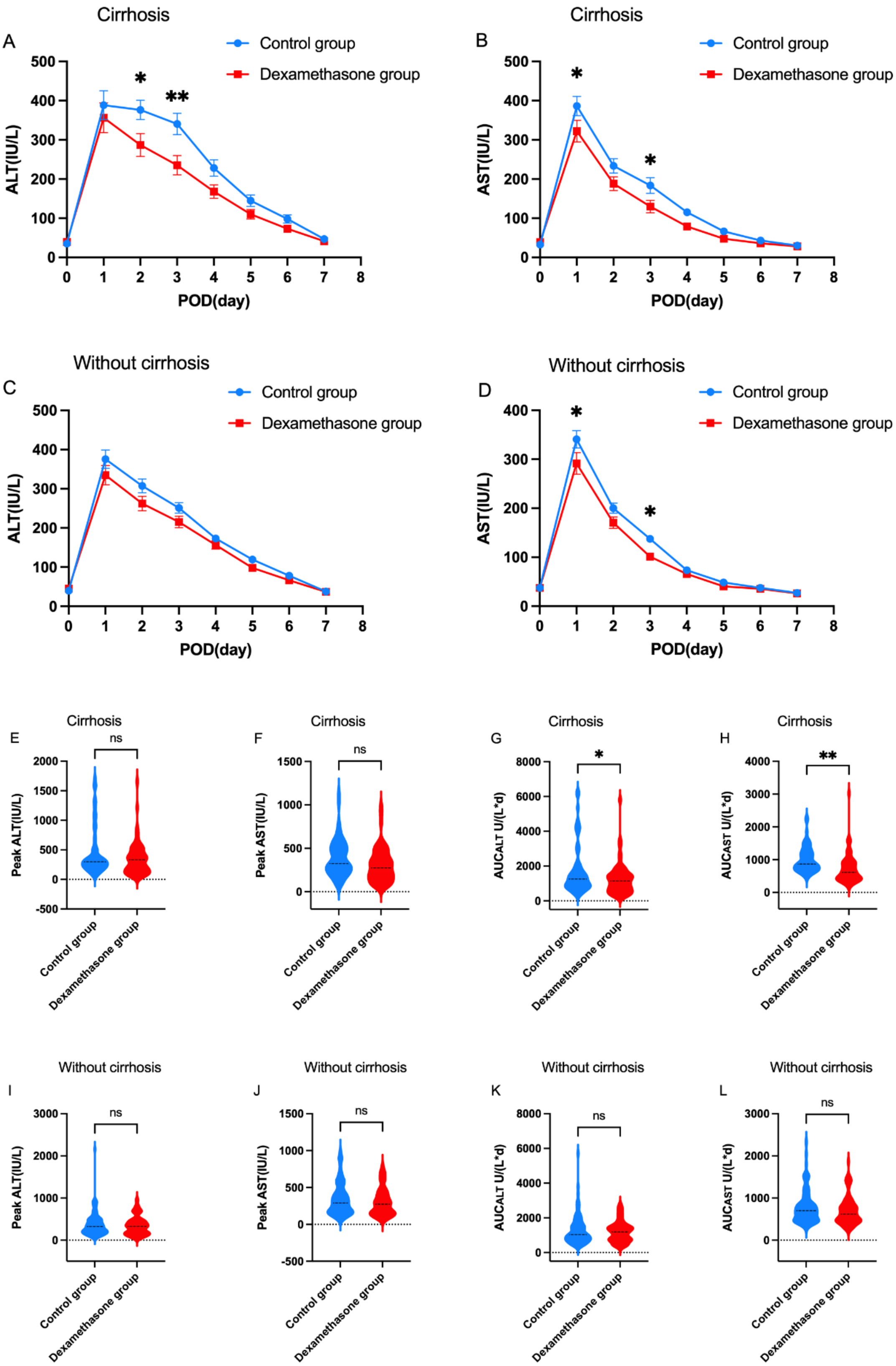

A
Cirrhosis
Control group
Dexamethasone group
ALT(IU/L)
POD(day)
*
**
B
Cirrhosis
Control group
Dexamethasone group
AST(IU/L)
POD(day)
*
*
C
Without cirrhosis
Control group
Dexamethasone group
ALT(IU/L)
POD(day)
D
Without cirrhosis
Control group
Dexamethasone group
AST(IU/L)
POD(day)
*
*
E
Cirrhosis
ns
Peak ALT(IU/L)
Control group
Dexamethasone group
F
Cirrhosis
ns
Peak AST(IU/L)
Control group
Dexamethasone group
G
Cirrhosis
*
AUCALT U/(L*d)
Control group
Dexamethasone group
H
Cirrhosis
**
AUCAST U/(L*d)
Control group
Dexamethasone group
I
Without cirrhosis
ns
Peak ALT(IU/L)
Control group
Dexamethasone group
J
Without cirrhosis
ns
Peak AST(IU/L)
Control group
Dexamethasone group
K
Without cirrhosis
ns
AUCALT U/(L*d)
Control group
Dexamethasone group
L
Without cirrhosis
ns
AUCAST U/(L*d)
Control group
Dexamethasone group

Supplementary Figure 2: Serial measurements of serum ALT, AST in subgroup of major hepatectomy (A-B) and minor hepatectomy (C-D) for the dexamethasone and control group. Data were presented as mean ± standard error. Violin plots showing the peak ALT, peak AST, AUC_{ALT} and AUC_{AST} levels in subgroup of major hepatectomy (E-H, respectively) and minor hepatectomy (I-L, respectively) for the dexamethasone and control group. Solid lines represented median value. ALT, alanine aminotransferase; AST, aspartate aminotransferase; AUC_{ALT}, area under the curve of alanine aminotransferase; AUC_{AST}, area under the curve of aspartate aminotransferase; POD, postoperative day. (*: $p<0.05$; ns: no significance)

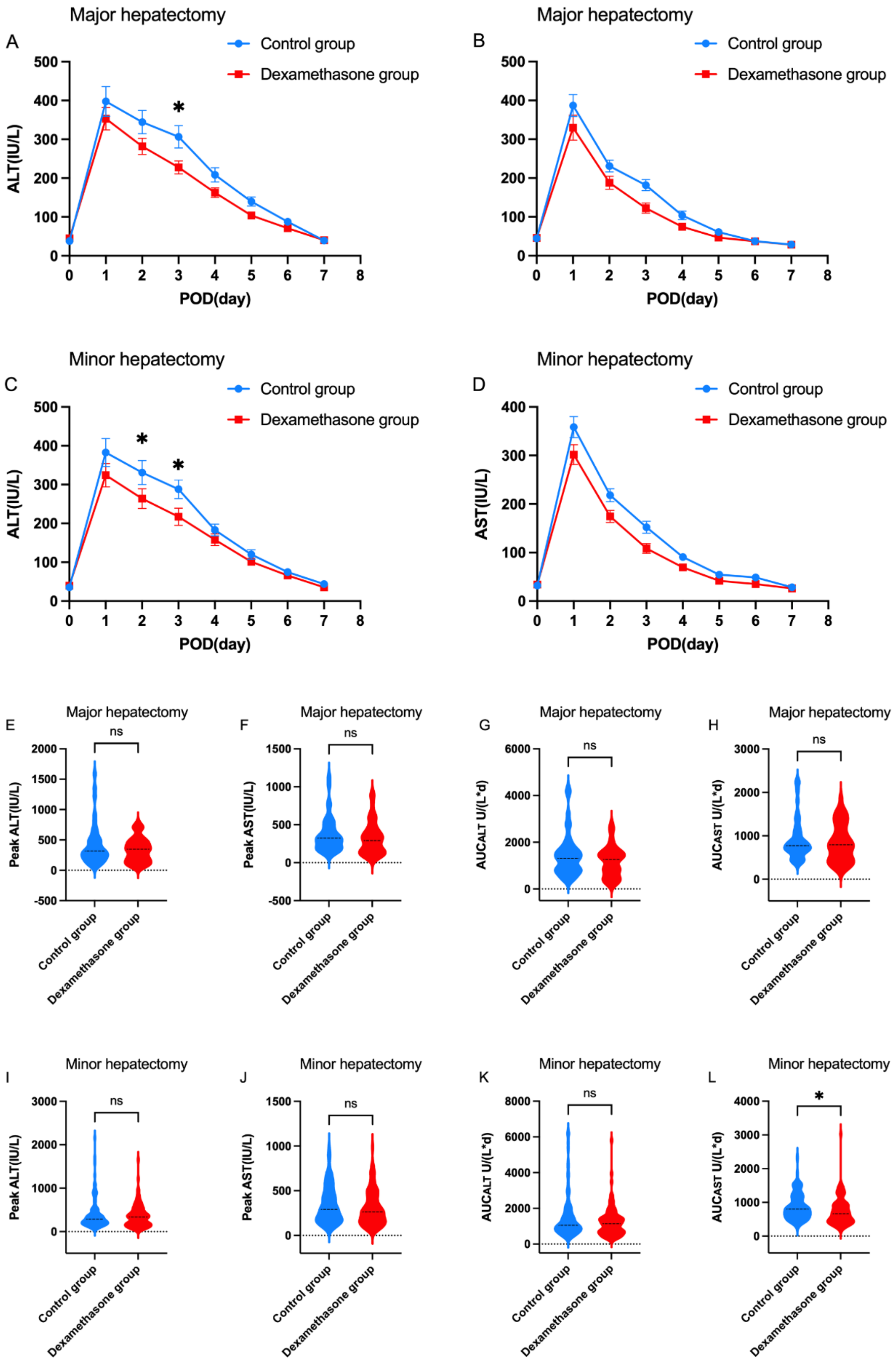

A
Major hepatectomy
Control group
Dexamethasone group
ALT(IU/L)
POD(day)
B
Major hepatectomy
Control group
Dexamethasone group
POD(day)
C
Minor hepatectomy
Control group
Dexamethasone group
ALT(IU/L)
POD(day)
D
Minor hepatectomy
Control group
Dexamethasone group
AST(IU/L)
POD(day)
E
Major hepatectomy
ns
Peak ALT(IU/L)
Control group
Dexamethasone group
F
Major hepatectomy
ns
Peak AST(IU/L)
G
Major hepatectomy
ns
AUCALT U/(L*d)
H
Major hepatectomy
ns
AUCAST U/(L*d)
I
Minor hepatectomy
ns
Peak ALT(IU/L)
J
Minor hepatectomy
ns
Peak AST(IU/L)
K
Minor hepatectomy
ns
AUCALT U/(L*d)
L
Minor hepatectomy
*
AUCAST U/(L*d)
